# Case Report: Effects of Multiple Seasons of Heavy Strength Training on Muscle Strength and Cycling Sprint Power in Elite Cyclists

**DOI:** 10.3389/fspor.2022.860685

**Published:** 2022-04-25

**Authors:** Bent R. Rønnestad

**Affiliations:** Section for Health and Exercise Physiology, Inland Norway University of Applied Sciences, Lillehammer, Norway

**Keywords:** case report, endurance, performance, cycling, resistance training

## Abstract

Sprint performance is critical for endurance performance in sports characterized by multiple accelerations like a cross-country Olympic mountain bike (XCO MTB) race. There are indications that 10–25 weeks of heavy strength training (HST) can improve cycling sprint power in cyclists. However, there is a lack of data on the effect of continuing HST across several seasons. In the first part of this case report, two elite cyclists performed HST across two preparatory periods (i.e., 1.5 years), while two others continued with endurance training only. HST induced a mean increase in leg press force and cycling sprint power of 16% after the first preparatory period (November to April), which was maintained during the competition period. After the next preparatory period a further increase from the first test was achieved (22 and 19%, respectively). The two cyclists with no HST had no changes in leg press force and cycling sprint power. The second part contains data from two of the cyclists from the first part. One of them continued with HST for 2 more years and achieved a continuous increase in leg press force during all four preparatory periods, ending up with a total increase of 44% after 3.5 years, while the development of cycling sprint power had more variation with an apparent plateau from the third to fourth preparatory periods, ending up with an improvement of 25%. The other cyclist did not perform HST in the first part but started with HST and performed this across the last two preparatory periods. After two preparatory periods with HST (i.e., 1.5 years), the increased leg press force and cycling sprint power were 24 and 22%, respectively, which was in the same range as the improvement observed after 1.5 years of HST in the first part of this case report. The present data extend previous short-term studies indicating that HST can give reasonable muscle strength improvements in elite cyclists across multiple preparatory periods. Furthermore, the present data indicate that HST adaptations can be maintained across multiple competition periods. Cycling sprint power seems to approximately follow the development of leg press performance.

## Introduction

There is growing evidence that 8–12 weeks of heavy strength training (HST) is beneficial for endurance performance in different Olympic endurance sports like running and cycling (e.g., Rønnestad and Mujika, 2014; Mujika et al., 2016; Blagrove et al., 2018). Endurance performance is, of course, affected by numerous variables, and in this big picture, HST is likely to play a minor role and is of less importance than, for example, endurance training (Rønnestad and Mujika, 2014). That being said, sprint performance and anaerobic capacity can be crucial for endurance performance, like during the final sprint toward the finish line or during a race in sports characterized by stochastic changes in exercise intensity and multiple accelerations like during an Olympic game of road race with a closed-circuit criterium style (Babault et al., 2018; Etxebarria et al., 2019), a cross-country Olympic mountain bike (XCO MTB) race (Granier et al., 2018), or cross-country skiing (Losnegard, 2019). Improved muscle strength and cycling sprint power are among adaptations that are rather specific to HST and, thus, less affected by endurance training. Therefore, it is expected that HST has been repeatedly observed to increase cycling sprint power in cyclists (Rønnestad et al., 2010a, 2015, 2017; Vikmoen et al., 2016; Beattie et al., 2017). However, latter studies have a duration ranging from 10 to 25 weeks, which, from a perspective of a 4-year Olympic cycle, is a short-term duration. There is a lack of data on the effect of continuing HST across several competition seasons and preparatory periods on elite cyclists.

This case study, therefore, presents muscle strength and cycling sprint power data from four elite XCO MTB cyclists with similar content of endurance training. Two cyclists decided to engage in HST across two preparatory periods (i.e., 1.5 years), while the two other cyclists continued with similar training to the two first cyclists except HST. Furthermore, after the first part, one of the two HST cyclists continued with HST for 2 more years, while one of the two who did not perform HST started with HST across the last two preparatory periods. Thus, this study shows the development of muscle strength and cycling sprint power in an elite cyclist who performed HST continuously for 3.5 years.

## Case Description

The participants were four male cyclists from the Norwegian national XCO MTB team who at baseline were tested for maximal oxygen uptake (VO_2max_; Oxycon Pro, mixing chamber, Erich Jaeger, Hoechberg, Germany) and mean power during the last minute of this test with 25W·min^−1^ increases until exhaustion (W_max_; Lode Excalibur Sport, Lode B. V., Groningen, Netherlands). Two of the cyclists initiated HST in addition to usual endurance training (E&S1: age: 19 years, height: 183 cm, body mass: 66.8 kg, VO_2max_: 83.4 mL·min^−1^·kg^−1^, and W_max_: 7.1 W·kg^−1^; E&S2: age: 19 years, height: 179 cm, body mass: 61.8 kg, VO_2max_: 80.6 mL·min^−1^·kg^−1^, and W_max_: 7.1 ·kg^−1^). The two other cyclists continued the usual endurance training (*E1*: age: 18 years, height: 183 cm, body mass: 75.3 kg, VO_2max_: 81.1 ml·min^−1^·kg^−1^, and W_max_: 7.1 W·kg^−1^; *E2*: age: 18 years, height: 180 cm, body mass: 62.8 kg, VO_2max_: 80.8 ml·min^−1^·kg^−1^, and W_max_: 7 W·kg^−1^). They all trained together, had the same coach, and competed in the World Cup, world championships, European championships, and national races.

## Training Intervention and Measured Variables

**Figure 1:**
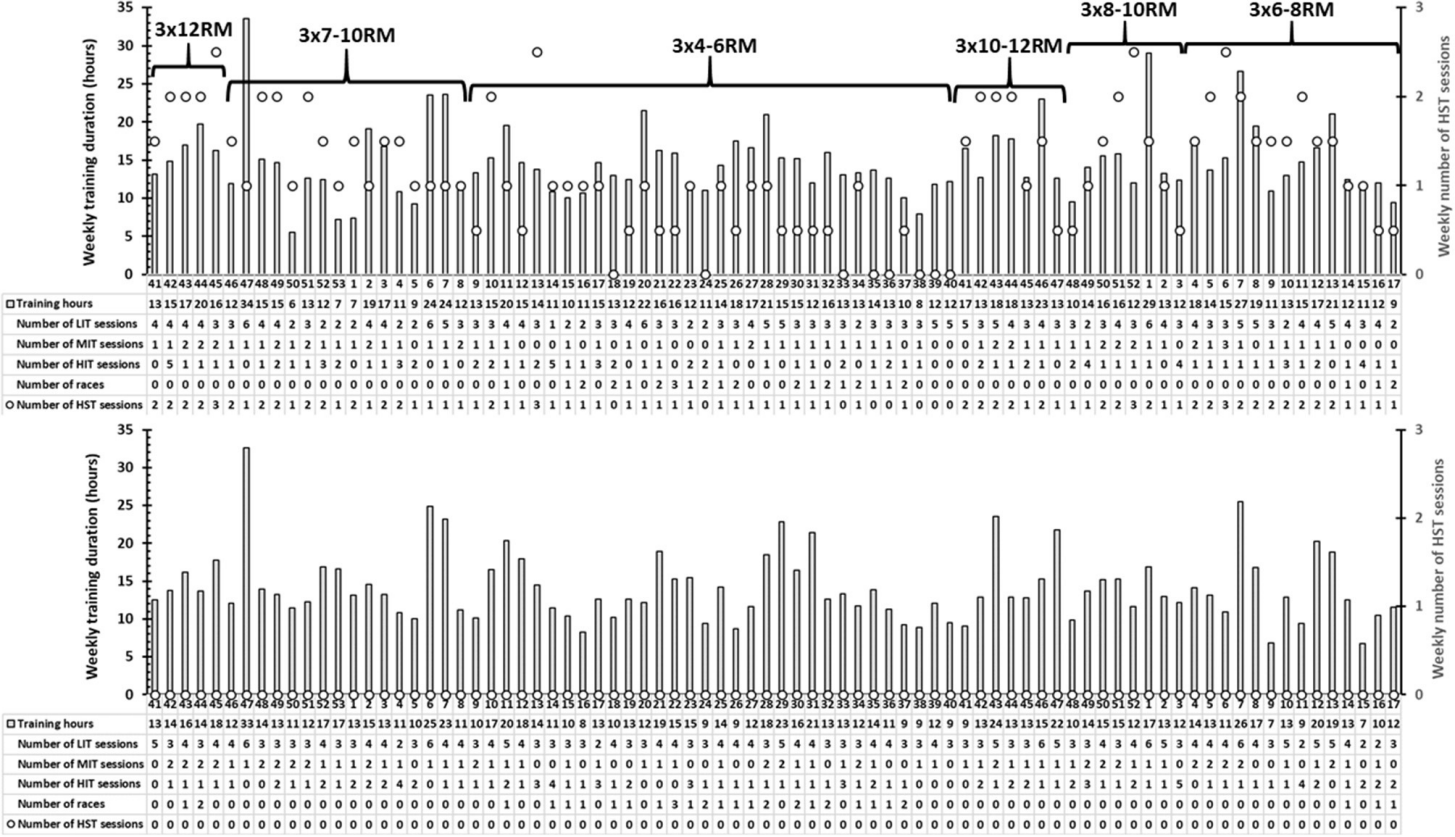
Mean weekly training duration (bars, y1-axis), mean weekly number of heavy strength training sessions (circles, y2-axis), weekly mean number of low-intensity training (LIT) sessions, moderate-intensity training (MIT) sessions, and high-intensity interval training (HIT) sessions, and distribution of sets and repetitions of the strength training for the two cyclists starting with heavy strength training (*EandS1* and *EandS2;* upper panel) and for the two cyclists continuing endurance training only (*E;* lower panel). Data start with the preparatory period *via* the competition period and the new preparatory period, and conclude at the start of the following competition period (i.e., 1.5 years).

*E&S1* and *E&S2* started with HST training in the beginning of the preparatory period in combination with endurance training. The HST followed the recommendations from previous studies on cyclists aiming at 2 weekly sessions for the development of muscular strength and one session every 7–10 days for maintenance with the strength training load adjusted according to the repetition maximum (RM) principle with a systematic variation between 4 and 12 RMs and 3 sets with ~2 min set pauses (Rønnestad and Mujika, 2014; Rønnestad et al., 2016). The strength exercises were focused on the lower-body and included half-squats in a Smith machine, leg presses with one foot at a time, one-legged hip flexions, and toe raises (Rønnestad et al., 2010b). The HST was performed with the intention of maximal acceleration during the concentric phase, while the eccentric phase was performed more slowly (Rønnestad et al., 2010b). Figure 1 depicts the mean weekly number of HST sessions and distribution of sets and repetitions of *E&S1* and *E&S2* across the first part of this case report starting with the preparatory period *via* the competition period and the new preparatory period, and concludes at the start of the following competition period (Figure 1). The endurance training for the two *E&S* and the two *E* cyclists is shown as mean weekly training duration as well as mean weekly number of low-intensity training (LIT) sessions, moderate-intensity training (MIT) sessions, and high-intensity training (HIT) sessions (Figure 1). The presentation of the endurance training was complex, since training registration varied during this long period of data sampling. Therefore, the session goal approach was used (Sylta et al., 2014). In cases where a long 4-h ride included 1 h of threshold exercise and the remaining at low intensity, 1 LIT and 1 MIT sessions were registered. The HST was usually performed as an afternoon session, 4–6 h after an LIT session.

**Figure 2:**
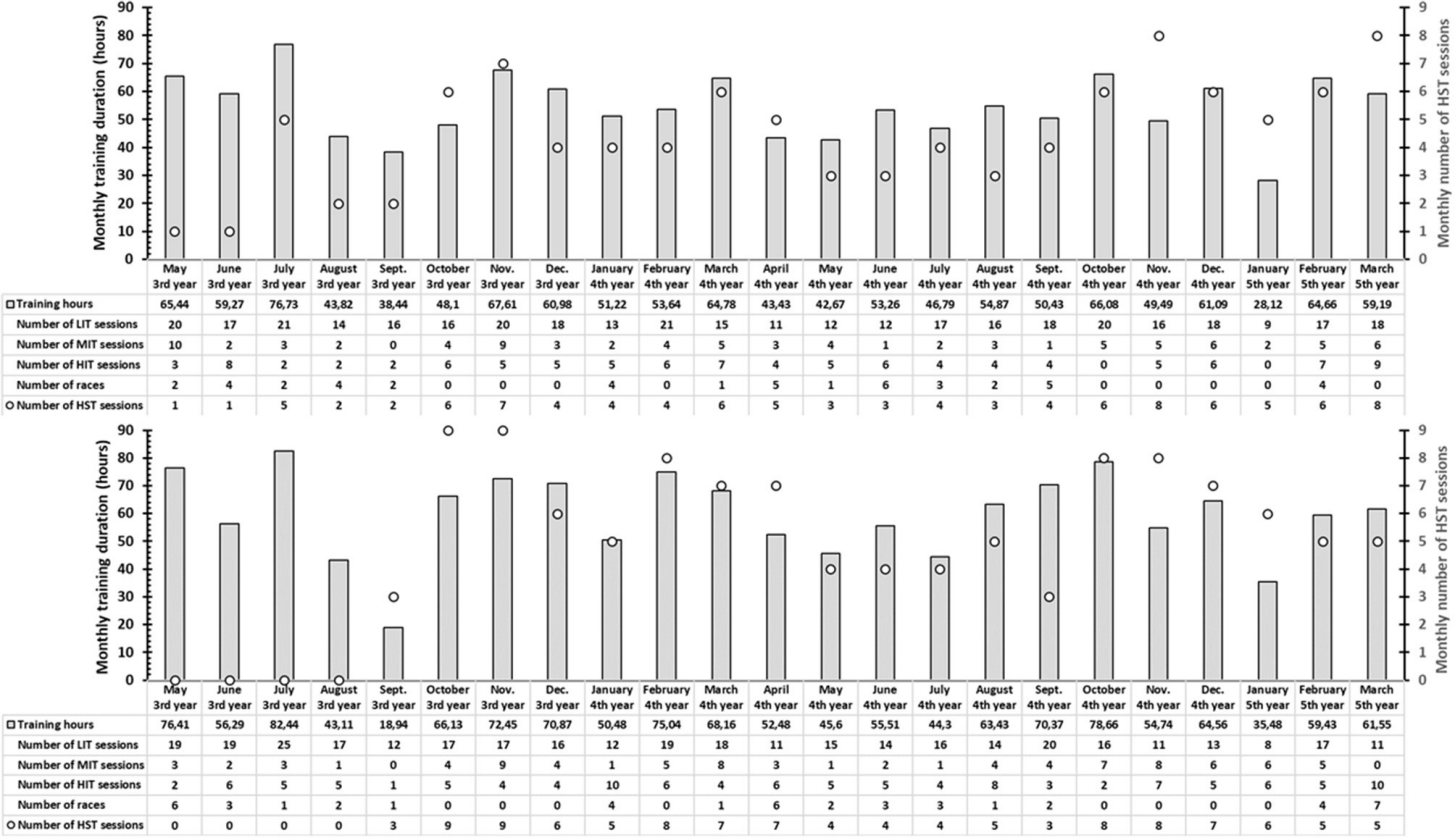
Monthly training duration (bars, y1-axis), monthly number of heavy strength training sessions (circles, y2-axis), monthly number of low-intensity training (LIT) sessions, moderate-intensity training (MIT) sessions, and high-intensity interval training (HIT) for the cyclist continuing with heavy strength training after the first part (*EandS1*; upper panel) and the cyclist who started with heavy strength training in the preparatory period after the first part (*E1* + *EandS*; lower panel). Data start with the competition period *via* the preparatory period, new competition period, and start of the last preparatory period, and conclude at the start of the following competition period (i.e., 2 years).

After the first part, lasting 1.5 years, one cyclist in each group did not get satisfying results in training/testing and thus dropped out of this data collection. The second part of this case report, therefore, presents data from *E&S1* who continued HST for 2 more years, and from *E1* who initiated HST when the next preparatory period started and continued this for 1.5 years (*E1* + *E&S*). The HST for both *E&S1* and *E1* + *E&S* in the second part followed the same guidelines as the first part and was, therefore, not repeated. The training data from the last 2 years are presented in Figure 2. Thus, the second part shows the development of leg press performance and 6-s cycling sprint power in a cyclist who performed HST continuously for 3.5 years (*E&S1*) and in a cyclist who only performed endurance training during the first 2 years and then performed HST during the last 1.5 years (*E1* + *E&S*).

All test days were preceded by 1 day of easy training and contained individual standardization for meal and caffeine consumption, temperature, testing time of day, warm-up, and test equipment adjustments. The testing started with a 10-min warm-up on an ergometer cycle at a rate of perceived exertion equal to 11–12 on a 6–20 scale (Borg, 1982) before a horizontal leg press peak force and peak power test was performed (Keiser AIR300 Leg Press; Keiser Corp., Fresno, CA, United States). The cyclists sat with knees flexed at approximately 90° and the hips flexed at approximately 45°, with the individual seating position being identical for all tests. Two warm-up repetitions were performed at ~40 kgf. The testing consisted of a single trial of 9–13 lifts with standardized increase in resistance (until failure) and standardized recovery periods between lifts (gradually increasing from 5 to 60 s), and loads starting at ~40 kgf and ending at ~250–350 kgf (depending on maximal strength). During all the lifts, the participants were instructed to exert force as fast as possible with maximal effort. The average concentric mechanical power and force of each lift were calculated with the manufacturer's software. Based on these calculations, peak power output and peak force were calculated as means of the two legs and used for data analysis. After a 5-min recovery and subsequent 10 min of cycling, including two submaximal sprints with 2-min recovery in between, two 6-s sprints (also 2-min recovery between them), one seated and one standing, were performed on a Lode Excalibur Sport (Lode B. V., Groningen, Netherlands) ergometer in Wingate mode. The load was calculated from the participant's total body mass and set to 0.8 Nm·kg^−1^ body mass. Mean power output for the two sprints was used for data analysis.

## Outcomes and Results

### First Part

**Figure 3:**
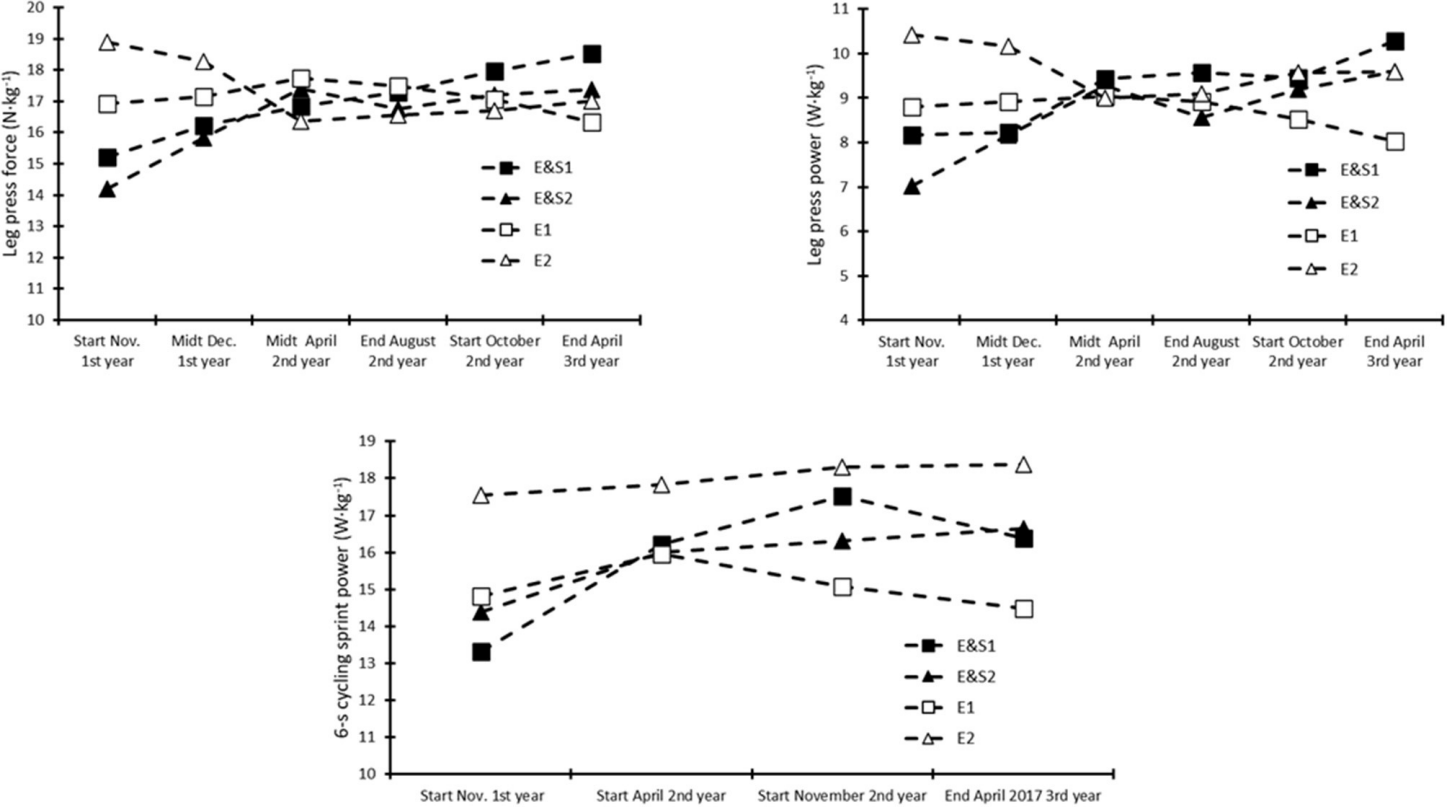
Leg press force (upper left panel), leg press power (upper right panel), and 6-s cycling sprint mean power (lower panel) of the two cyclists who started with heavy strength training (*EandS*) and the two cyclists who continued endurance training only (*E*). Data start with the preparatory period (November) *via* the competition period (starting in April) and the new preparatory period (starting in October) and conclude at the start of the following competition period (April), altogether lasting for 1.5 years.

During the first preparatory period (November-April) *E&S1* and *E&S2* increased leg press force by 11 and 22%, respectively, leg press power by 15 and 32%, respectively, and, more or less, maintained leg press force (14 and 18%, respectively) and leg press power (17 and 22%, respectively) during the competition period until the end of August (Figure 3). Following the second preparatory period, in April, the leg press force of both *E&S1* and *E&S2* increased by 22% from the first test, while leg press power change was 26 and 37%, respectively (Figure 3). During the 1.5 years, *E1* and *E2* had a slight reduction in leg press force (−3 and −10%, respectively) and leg press power (−9 and −8%, respectively; Figure 3). Mean power output during the 6-s cycling sprint increased in both *E&S1* and *E&S2* during the first preparatory period (22 and 11%, respectively), while that in *E&S1* had little change during the second preparatory period, ending up with a 23% change from the first test, while that in *E&S2* ended the second preparatory period slightly higher than that in the first (16%; Figure 3). During the 1.5 years, *E1* and *E2* had only small changes in 6-s cycling sprint power output (−2 and 4%, respectively; Figure 3).

### Second Part

**Figure 4:**
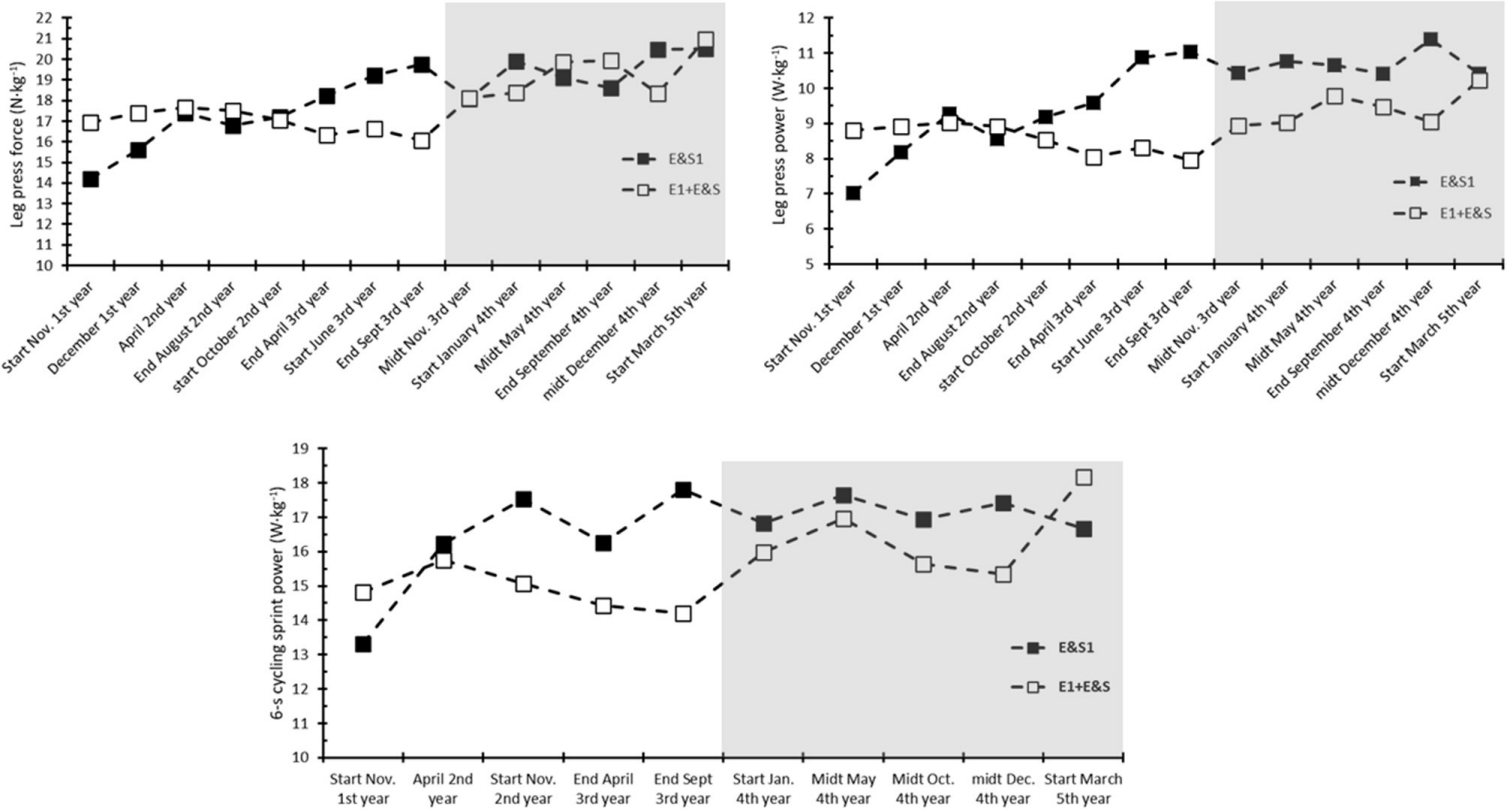
Leg press force (upper left panel), leg press power (upper right panel), and 6-s cycling sprint mean power (lower panel) of the cyclist who performed heavy strength training during both the first and the second parts (*EandS1*) and the cyclist who started with heavy strength training in the preparatory period after the first part (*E1* + *EandS*; gray area indicates the strength training period for *E1* + *EandS*). Data start with the first test during the first part and conclude with the last test during the second part, altogether lasting for 3.5 years.

After the second competition period, *E&S1* had an increase in leg press force and power of 39 and 57%, respectively, from the first test, while *E1* had a reduction of 5 and 10%, respectively. During the subsequent preparatory period when *E1*+*E&S* started with the HST, *E&S1* maintained the increase in leg press force and power in January (40 and 53%, respectively), while *E1*+*E&S* had an increase from the first test of 9 and 3%, respectively. In May, in the following competition season, change in force and power from the first test for *E&S1* was 34 and 52%, respectively, and for *E1* + *E&S* it was 17 and 11%, respectively, which was maintained until the end of the competition period in September (31 and 48% vs. 18 and 7%, respectively). After the last preparatory period, *E&S1* and *E1* + *E&S* had increased leg press force by 44 and 24%, respectively, and leg press power by 48 and 16%, respectively (Figure 4).

After the second competition period, *E&S1* had an increase in mean power output during the 6-s cycling sprint of 33%, while *E1* + *E&S* had a reduction of 4% from the first test. During the two next preparatory periods, there were only small changes in the sprint power of *E&S1*, ending up with 25% improvement from the last test after 3.5 years of HST. During the first preparatory period with HST, *E1*+*E&S* increased sprint power by 14% from the first test, with further improvement of 23% increase at the end of the second preparatory period (Figure 4).

## Discussion

This case report provides a unique 3.5-year example of the effect of combining endurance and HST on leg press force and power as well 6-s cycling sprint mean power output in an elite cyclist. The first part of this case included two control cyclists that simply continued their endurance training for 1.5 years and two cyclists who started with HST.

### First Part

During the first preparatory period (~5 months), the HST cyclists had a mean improvement in leg press force and power of 16 and 23%, respectively and, thus, reached the same leg press values as the control cyclists when the competition period started. This initial improvement is in accordance with previous studies that observed 10–20% increase in maximal strength after 20–25 weeks of strength training in cyclists (Rønnestad et al., 2015; Beattie et al., 2017). Cessation of HST during the first 8 weeks of the competition period has previously reduced gain in muscle strength and cycling sprint power achieved during the preparatory period (Rønnestad et al., 2016). However, by performing regular HST, with no more than 3 weeks in a row without HST, the strength training cyclists were able to maintain their leg press strength and power during the competition period (April to September). Maintenance of strength training adaptations by approximately 1 HST session every 10 days is in accordance with previous studies investigating the maintenance effect for 13–15 weeks (Rønnestad et al., 2010a, 2015). Interestingly, after maintaining the muscle strength improvements during the competition period, the mean values of leg press force and power continued to increase during the second preparatory period, ending with a total increase of 22 and 31%, respectively. Thus, after continuous HST for 1.5 years, the two *E&S* cyclists had changed from having the lowest muscular strength to having the highest muscular strength among the four cyclists in this case report. Simply continuing the endurance training had no effect or even possibly had a slightly negative effect on muscular force. Changes in 6-s cycling sprint power followed the changes in leg press variables, where the *E&S* cyclists had a mean increase of 16% after the first preparatory period. This improvement in cycling sprint power is somewhat larger than that observed after 20–25 weeks of HST (Rønnestad et al., 2015; Beattie et al., 2017). Furthermore, 6-s cycling sprint mean power had a slight increase during the second preparatory period and ended with a total mean improvement of 22% after 1.5-year HST, while the control cyclists with no HST achieved a total change of 1%. Sprint power is mainly dependent on the size of the involved muscle mass and maximal leg strength (Izquierdo et al., 2004); thus, the observed changes in the 6-s cycling sprint power of cyclists *E&S* and *E* are likely related to the observed change (or lack of change) in their leg press performance.

### Second Part

The big picture in the development of *E&S1'*s leg press force is that it increased during the four preparatory periods and was maintained during the competition periods, leading to a 22% increase after the first preparatory period, 28% increase after the second period, 35% increase after the third period, and 44% increase after the fourth period. Leg press power and cycling sprint power had a bit more variation in development, with an apparent plateau from the third to the fourth preparatory period and ending up with an improvement of 48 and 25%, respectively. Despite the usual MTB XCO training and the competition including numerous short high-intensity sprints (Granier et al., 2018), it is interesting to see that the present elite cyclists seemed to have not developed leg press performance and 6-s cycling sprint power during the 1.5–2 years of training (while going from 18 to 20 years of age). However, when *E1* + *E&S* started the HST, he clearly improved in both leg press performance and 6-s cycling sprint power during the preparatory period. After his second preparatory period (i.e., 1.5 years of HST), leg press force and power and 6-s cycling sprint power were increased by 24, 16, and 22%, which was quite similar to the response of *E&S1* and *E&S2* after similar time of HST. The data from *E1* + *E&S* (and *E&S1* and *E&S2*) indicate that HST is a potent stimulus for increasing cycling sprint power.

## Subject Perspective

The cyclists reported more heaviness and soreness in the legs in the beginning of the HST, and that was the reason why HST was always initiated at the beginning of the preparatory period, when the endurance training was not so important. This negative effect of the strength training was also most pronounced in periods with 10–12 RM loading (in the first part of every preparatory period). Therefore, lower numbers of RM were applied during the competition period. During the competition period, the cyclists sometimes experienced challenges in performing the HST because of combination of tight race schedules and travel. However, the cyclists experienced that the HST improved their performance, not only in sprints, but also in their strength felt throughout the entire race. However, this case report had no data that can verify this feeling, but previous studies support that HST can improve lactate threshold power output, W_max_, and 5- to 40-min maximal power output without negatively affecting VO_2max_ (reviewed in Rønnestad and Mujika, 2014). Future studies should investigate the effect of multiple years of HST on muscle hypertrophy, endurance performance determining variables, and endurance performance utilizing an appropriate sample size of well-trained cyclists.

## Limitations

A limitation is the lack of a specific measure of cycling performance beyond the simple 6-s cycling sprint on an ergometer bike. One may question the usefulness of HST in MTB XCO cyclists if no endurance performance or endurance performance determinants are measured. However, sprint performance and anaerobic capacity can be important for endurance performance, like getting a good position after the start in a MTB XCO race, when they are entering the single track (saving energy, reduce risk of crashes and accidents). Furthermore, this ability is crucial during the final sprint toward the finish line or during a race in sports characterized by stochastic changes in exercise intensity and multiple accelerations like during an Olympic game of road race with a closed circuit criterium-style (Babault et al., 2018; Etxebarria et al., 2019) or an XCO MTB race (Granier et al., 2018). The present case report is limited to a practical example on how HST can be implemented across multiple years in the training of elite cyclists.

## Conclusion

The present data extend previous short-term studies indicating that 2 weekly sessions of HST gives reasonable strength improvements when the training load varies between 4 and 12 RMs in elite cyclists, and this seems to also hold true across multiple preparatory periods. Furthermore, HST adaptations seems to be maintained across multiple competition periods with 1 HST session approximately every 10 days. Cycling sprint power seems to approximately follow the development of leg press performance.

## Data Availability Statement

The datasets generated for this study are available on request to the corresponding author. The data are not publicly available due to their containing information that could compromise the privacy of the research participants.

## Ethics Statement

Ethical review and approval was not required for the study on human participants in accordance with the local legislation and institutional requirements. The patients/participants provided their written informed consent to participate in this study. Written informed consent was obtained from the individual(s) for the publication of any potentially identifiable images or data included in this article.

## Author Contributions

BRR collected the data and wrote the manuscript.

## Conflict of Interest

The author declares that the research was conducted in the absence of any commercial or financial relationships that could be construed as a potential conflict of interest.

## Publisher's Note

All claims expressed in this article are solely those of the authors and do not necessarily represent those of their affiliated organizations, or those of the publisher, the editors and the reviewers. Any product that may be evaluated in this article, or claim that may be made by its manufacturer, is not guaranteed or endorsed by the publisher.
